# Leguminous Food

**Published:** 1876-03

**Authors:** 


### LEGUMINOUS FOOD.

The principal leguminous food in the United States are the bean and pea. The lentil is raised to some extent, but is not largely cultivated among us. The question is asked will they sustain active life. We are told that Liebig has stated that they are too poor in phosphates to supply the osseous waste, and that they are more shadowy than substantial, filling up and satisfying hunger without affording nourishment.

So far as the phosphatic element is concerned, we believe the distinguished German chemist is correct. The legumes are not rich in phosphate, although they are not wholly without this very important element. But they contain albumenous, nitrogenous, and oleagenous elements in large degree, and are, therefore, givers of heat and builders up of muscular and nervous waste. They have great power as muscle-sustainers, and we can not think Liebig really intended to convey the idea that because poor in phosphates they were inadaquate to build up and supply the waste of tissue.

Our belief is that the man who labors with his hands can live well and keep well, other things being equal, on good bread made from cereals not poisoned by grinding, nor robbed of gluten and phosphate by bolting, and not destroyed by bad cooking, and well-cooked beans or peas. Experience proves this. In the late war there was any amount of hard marching and desperate fighting performed on a bean and wheat diet.

Beans are a very valuable food. Persisted in we have known them to cure many cases of scrofulous taint accompanied by disfiguring eruptions. The contained oil, not unlike castor oil in character, induces a gentle activity of the alimentary canal, and the effects of the blood-poison disappears in due time.

But with beans and peas, cooking is everything. As ordinarily brought to the table, they are utterly unfit for a human stomach. They are almost never cooked enough. They are heavily freighted with salt and pork and grease. The pork is perhaps stale or ill-fed and rank. It gives off its excess of salt in the cooking, and the beans absorb it. It exudes grease and the beans are saturated with it. The pot of baked beans is often a mess indigestible save by the strongest stomachs, and certain to destroy the solvent power of even the strongest. There is a close-grained tenaceous hull upon the bean and pea, composed of cellulose or woody fiber, which takes high heat, long continued, to dissolve. This shell can not be digested until dissolved. The cooking process must do this, or the mass must be strained or sifted. If beans are cooked in a double boiler—a boiler having a water-jacket, as they should be—they can be kept on the fire for days without burning. Baking is not the best mode of cooking beans and peas. They need water and a good deal of it. Bean soup is the best bean-food. It should be made thin, boiled long, and strained from its useless, irritating, and flatulent hulls. When cold it will form a solid jelly. Eaten with cream it is delicious.

Somebody should devise a method of removing the irritating hull, and should afterward reduce the bean and pea to a flour. This would fit it for quick preparations for the table. We believe that the cold air attrition process, which is doing such a grand work for our wheat by reducing the whole kernel to a fine powder, will be found applicable to the légumes as well, and we hope the Health Food Company will cause the attempt to be made. Whenever dried beans and peas are hulled and powdered, they will become very popular food. Until then, with our careless and lazy cooks, these valuable articles will be neglected, because only iron stomachs with gastric juice, having nearly the solvent power of *aqua fortis*, can digest them. For the present let those who eat these foods see that they are literally "cooked to death;" that is, until the outer coat has undergone absolute dissolution.

